# Influencing factors of pain management knowledge and attitudes among emergency nurses in China: a cross-sectional study

**DOI:** 10.3389/fpubh.2026.1815532

**Published:** 2026-04-01

**Authors:** Qin Jiang, Junjie Zhao, Rong Wu

**Affiliations:** 1 Nursing Department, Sir Run Run Shaw Hospital, Zhejiang University School of Medicine, Hangzhou, China; 2 Emergency and Critical Care Center, Department of Emergency Medicine, Zhejiang Provincial People's Hospital (Affiliated People's Hospital), Hangzhou Medical College, Hangzhou, China

**Keywords:** emergency, cross-sectional study, attitude, pain management, knowledge, nurse

## Abstract

**Background/Objective:**

Effective pain management in emergency department depends on nurses having both adequate knowledge and a positive attitude. This study aims to evaluate the factors influencing the knowledge and attitude toward pain management among emergency department nurses.

**Methods:**

In this cross-sectional study, a survey was conducted among 345 nurses working in the emergency department in China using the Wenjuanxin platform. Data were collected using a demographic information checklist and two standardized questionnaires: the Emergency Room Staff Pain Management Knowledge Questionnaire (ER-SPMKQ), and the Nurses’ Attitude Survey (NAS). The aim of the study was to investigate the correlation between emergency department nurses’ pain management knowledge, their attitudes, and baseline characteristics.

**Results:**

A total of 345 nurses from the emergency department were included in the study. The mean scores for the ER-SPMKQ (Cronbach’s *α* = 0.716; KMO = 0.751, *χ*^2^ = 1293.854, *p* < 0.001) and NAS (Cronbach’s *α* = 0.719; KMO = 0.839, *χ*^2^ = 2921.131, *p* < 0.001) were 69.94 ± 17.67 and 62.86 ± 5.59, respectively. Among the participants, 42.90% had good knowledge, while 12.79% exhibited a highly positive attitude, and 86.67% demonstrated a moderate attitude toward pain management. Nurses with a Bachelor’s degree or higher, as well as those working in the emergency intensive care unit (EICU), displayed higher levels of knowledge (all *p* < 0.05). Additionally, nurses with higher educational backgrounds also exhibited more favorable attitudes toward pain management (*p* = 0.039).

**Conclusion:**

Although nurses in the emergency department have a favorable attitude toward pain management, there remains a gap in their knowledge of pain management. Continuous training and organized workshops are needed to further enhance their ability to manage pain in patients. Nurses with higher levels of education and those working in the EICU demonstrate a more profound understanding of pain management. Moreover, nurses with higher educational backgrounds also exhibit more favorable attitudes toward pain management.

## Introduction

Severe injuries or critical illnesses are common causes for individuals to seek emergency medical attention (1). Over one-third of these patients report experiencing moderate to severe pain upon arrival at the emergency department (2). However, despite being highly prevalent in emergency departments, pain is frequently underrecognized and undertreated (3, 4). Inadequate pain management in patients can result in a range of adverse health outcomes, including decreased patient satisfaction and an elevated risk of developing chronic pain (5). Therefore, a precise and comprehensive assessment is crucial for ensuring effective pain management (6).

Pain is a multidimensional phenomenon shaped by biological mechanisms, psychological states, and sociocultural contexts, and it may not be sufficiently represented by the widely used 0–10 numerical pain scale (7). Emergency nurses frequently encounter considerable variations in how patients express pain during routine care, often relying on observable behaviors to assess the severity of pain (8). This dependency on visible cues can result in inconsistencies in pain management, as the quality of care is directly affected by these subjective interpretations. Moreover, nurses are pivotal in managing pain by determining the timing for medication administration and selecting the most appropriate analgesics when multiple options are available (1). However, studies suggest that several factors may influence nurses’ ability to manage patients’ pain, including inadequate pain management training, insufficient education on opioid use, and negative attitudes towards pain treatment. These factors complicate the implementation of effective pain management strategies (1, 5, 9–11). Additionally, limited studies also highlight a significant gap in nurses’ knowledge and attitudes regarding pain management, with the absence of formal training in pain assessment during academic education being a key contributing factor (12, 13).

For addressing these barriers is essential for improving pain management practices and enhancing patient care outcomes. Therefore, researchers developed assessment tools to evaluate nurses’ knowledge and attitudes toward pain management (14). Several assessment tools, such as the Knowledge and Attitudes Survey Regarding Pain (KASRP), have been developed to evaluate nurses’ understanding of pain management, and have already been applied in China (15). However, the KASRP were originally developed for general clinical environments and may not fully capture the specific challenges and decision-making processes involved in emergency care.

In response to this gap, Wu et al. (16) developed the Emergency Room–Specific Pain Management Knowledge Questionnaire (ER-SPMKQ) to assess acute-care nurses’ pain management knowledge within the context of emergency settings. The ER-SPMKQ is a 25-item questionnaire that covers key domains such as pain assessment, pharmacological and non-pharmacological interventions, and the use of opioids in emergency scenarios. It was specifically designed to reflect the fast-paced and complex nature of emergency care, making it a suitable tool for evaluating emergency nurses’ pain management competencies.

Although the ER-SPMKQ was specifically designed for emergency care settings and has demonstrated good content validity and internal consistency, its use has been relatively limited, with few studies validating its applicability across different cultural and clinical contexts. Therefore, in this study, we adopted the ER-SPMKQ in combination with the Nurses’ Attitude Survey (NAS) to investigate the influencing factors of pain management knowledge and attitudes among emergency nurses in China.

## Methods

### Study aim

The primary aim of this study was to assess the knowledge and attitudes toward pain management among registered nurses working in the emergency departments of four hospitals in China. The secondary objective was to identify the correlation between nurses’ baseline characteristics and their knowledge and attitudes toward pain management.

### Participants and setting

This cross-sectional study utilized convenience sampling to conduct a questionnaire survey among emergency nurses at four hospitals in China. The inclusion criteria were as follows: (1) possession of vocational qualification certificates and employment in a hospital setting; (2) absence of dyslexia and ability to communicate fluently in Mandarin; (3) willingness to participate in this study. The exclusion criteria were as follows: (1) less than 6 months of work experience; (2) nurses not directly involved in clinical nursing care in the emergency department (e.g., administrative staff, research personnel, or trainees without independent clinical responsibilities); (3) incomplete questionnaires. The questionnaires were distributed via the Wenjuanxing platform. After excluding 70 nurses from non-clinical roles and 30 incomplete questionnaires, a total of 345 valid samples were obtained.

The sample size required for this study was calculated using the pwr package in R software, with a predetermined effect size of *f*^2^ = 0.02, statistical power of 1 – *β* = 0.8, and a significance level of *α* = 0.05. The calculation, assuming seven predictors, indicated that a minimum of 148 participants was required. The actual sample size for this study was 345 participants, which exceeds the required minimum and is sufficient to detect the expected effects in the analysis.

### Instruments

Data for this study were collected using three different instruments. The first was a demographic questionnaire that asked participants to self-report details such as age, gender, education level, pain management education experience, and nursing qualifications. Additionally, we utilized the ER-SPMKQ and NAS.

The ER-SPMKQ was developed by Wu and Lai in 2016 based on previous literature, specifically designed to be more applicable to pain management knowledge for emergency department nurses in China (16). The instrument includes: (1) Basic demographic information of emergency medical staff, covering 20 items such as gender, education level, age, job title, professional experience, hospital level, pain education training, pain experience, patient condition, pain assessment scales, and pain management guidelines; (2) A 25-item Pain Management Knowledge Scale for emergency medical staff, covering three main categories: pain assessment, pain management, and medication knowledge. The questionnaire uses multiple-choice questions, with 4 points awarded for a correct answer and 0 points for an incorrect one. A perfect score of 100 points is awarded for all correct answers and those who scored above the sample mean were classified as having relatively higher knowledge. This approach was adopted as a pragmatic method due to the absence of universally established or validated cut-off values for the ER-SPMKQ in the existing literature.

On the other hand, the NAS questionnaires were developed in the year 2000 by McMillan to assess nurses’ knowledge and attitudes concerning pain management (14). This tool consists of 25 items, each rated on a 4-point Likert scale, with a total score range from 25 to 100. For positively phrased statements, responses are scored as follows: “completely disagree” = 1, “disagree” = 2, “agree” = 3, and “completely agree” = 4. In contrast, for negatively phrased statements, the scoring is reversed. A score of 70% or higher indicates a highly positive attitude, while scores between 50 and 69% reflect a moderate attitude. A score below 50% is interpreted as a poor or negative attitude toward pain management.

### Data collection

The questionnaire was distributed among nursing personnel with approval from the Ethics and Research Committee during the period from December 2024 to January 2025, using the Wenjuanxing platform to collect data. Multiple reminders were sent to encourage responses and submission of the completed questionnaire.

### Statistical analysis

Data were statistically analyzed using SPSS (version 26; IBM Corp., Armonk, NY, USA) and R software (version 4.4.1; R Foundation for Statistical Computing, Vienna, Austria). Figures were created using GraphPad Prism (version 10; GraphPad Software, San Diego, CA, USA) and OriginPro (version 2024b; OriginLab Corp., Northampton, MA, USA). To ensure the normality of the distribution of quantitative data for participants, the Kolmogorov–Smirnov test was employed. Descriptive statistics, such as mean ± standard deviation (SD) and frequency (%), were utilized to present the knowledge and attitude to pain management in emergency department among nurses. In this study, inferential statistical analyses were conducted, utilizing Spearman’s correlation coefficient test to elucidate the relationships among knowledge and attitude, toward pain management in emergency department. Furthermore, these statistical methods were employed to discern correlation between nurses’ knowledge and attitudes relative to their demographic characteristics. The Mann–Whitney (or *t*-test) and Kruskal–Wallis tests were applied to assess differences based on sociodemographic characteristics. The internal consistency reliability of the two validated questionnaires was assessed using Cronbach’s alpha coefficient. Construct validity was evaluated using the Kaiser–Meyer–Olkin (KMO) measure and Bartlett’s test of sphericity. Since both instruments have been previously validated in the literature, no further exploratory or confirmatory factor analysis was conducted. The significance level for all tests was set at less than 0.05.

## Results

### Participants’ characteristics

A total of 345 participants completed the survey, with the following demographic and professional characteristics (Table 1): the majority of participants were aged between 26 and 30 years (46.38%), followed by those aged ≥31 years (34.78%) and ≤25 years (18.84%), with an average age of 28.36 ± 6.02 years. Most participants were female (85%), while males represented 15%. A large proportion of participants (82.03%) had less than 5 years of experience in the emergency department, while 17.97% had 5 years or more of experience. The predominant proportion of nurses (82.90%) possessed a bachelor’s degree. The nursing professional levels were distributed as follows: N0 (10.43%), N1 (15.65%), N2 (17.39%), N3 (30.43%), and N4 (26.09%). In terms of working units, 76.81% worked in the emergency room and 23.19% in the EICU. Regarding professional pain management training in the past 5 years, 94.20% of participants reported having received such training, while 5.80% had not. Table 1 shows the baseline characteristics of emergency nurses and its correlation with participants’ knowledge and attitude to pain management.

**Table 1:** Participant characteristics of the sample (*n* = 345).

Variables	Total (*n*, %)	Knowledge	*p-*value	Attitude	*p-*value
Age (year)					
≤25	65 (18.84)	70.28 ± 19.03	0.888	63.11 ± 5.94	0.853
26–30	160 (46.38)	70.20 ± 17.12		62.61 ± 5.61	
≥31	120 (34.78)	69.40 ± 17.79		63.07 ± 5.41	
Gender					
Female	292 (84.64)	69.95 ± 17.29	0.882	62.78 ± 5.61	0.481
Male	53 (15.36)	69.89 ± 19.84		63.32 ± 5.51	
Work experience in emergency department (year)					
<5 years	283 (82.03)	71.45 ± 18.10	0.175	63.21 ± 5.71	0.295
≥5 years	62 (17.97)	68.63 ± 17.24		62.56 ± 5.48	
Educational level					
Diploma	51 (14.78)	66.04 ± 19.86	0.023	61.31 ± 5.53	0.039
Bachelor’s Degree	286 (82.90)	70.49 ± 17.37		63.07 ± 5.61	
Master’s Degree	8 (2.32)	75.00 ± 9.50		65.38 ± 3.66	
Nursing professional level					
N0	36 (10.43)	70.89 ± 17.65	0.368	62.03 ± 5.30	0.332
N1	54 (15.65)	67.11 ± 18.37		63.70 ± 5.60	
N2	60 (17.39)	70.27 ± 20.13		63.67 ± 5.34	
N3	105 (30.43)	68.95 ± 15.61		62.66 ± 5.80	
N4	90 (26.09)	72.18 ± 17.87		62.39 ± 5.61	
Working unit					
EICU	80 (23.19)	73.65 ± 14.48	0.001	63.19 ± 6.02	0.487
Emergency room	265 (76.81)	68.82 ± 18.41		62.76 ± 5.47	
Professional pain management training in past 5 years					
Yes	325 (94.20)	70.35 ± 17.74	0.104	62.82 ± 5.65	0.584
No	20 (5.80)	65.14 ± 16.51		63.29 ± 4.93	

Group differences were assessed using Mann–Whitney *U* (or *t*-test) and Kruskal–Wallis tests, based on the distribution and number of groups.

### Reliability and validity analysis

The internal consistency of the two questionnaires was acceptable, with Cronbach’s *α* coefficients of 0.716 for the ER-SPMKQ and 0.719 for the NAS. Construct validity was supported by the KMO values (0.751 for ER-SPMKQ and 0.839 for NAS) and the results of Bartlett’s tests of sphericity (*χ*^2^ = 1293.854, *p* < 0.001; *χ*^2^ = 2921.131, *p* < 0.001, respectively). As both questionnaires have been previously validated, no further exploratory factor analysis was performed.

### Knowledge scores

**Figure 1:**
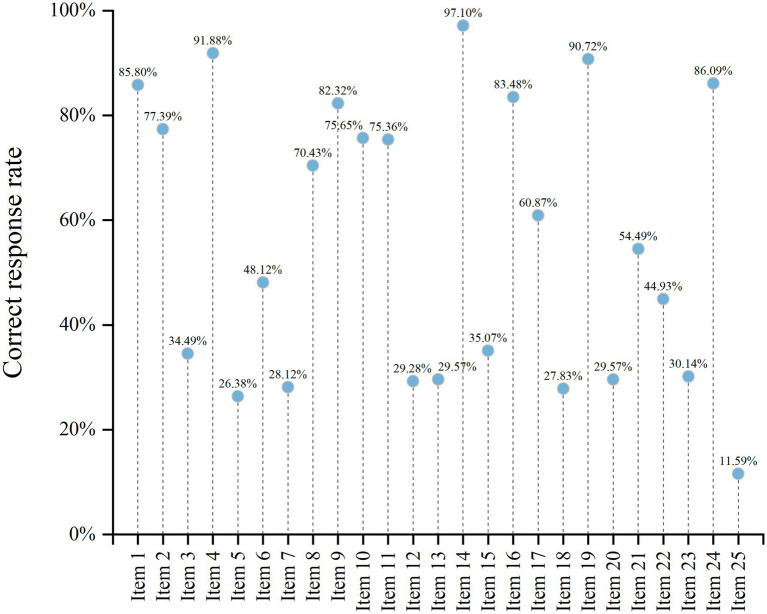
Correct response rates on Emergency Room Staffs’ Pain Management Knowledge Questionnaire. Detailed content of the questionnaire is provided in Supplementary file 1.

The average score on the ER-SPMKQ was 69.94 ± 17.67 (out of 100). Gender, age, nursing professional level, work experience in the emergency department, and professional pain management training in the past 5 years were not significantly associated with knowledge scores (all *p* > 0.05). In contrast, educational level (*p* = 0.023) and working unit (*p* = 0.001) showed statistically significant differences. A detailed breakdown of item responses is shown in Figure 1.

### Attitude scores

**Figure 2:**
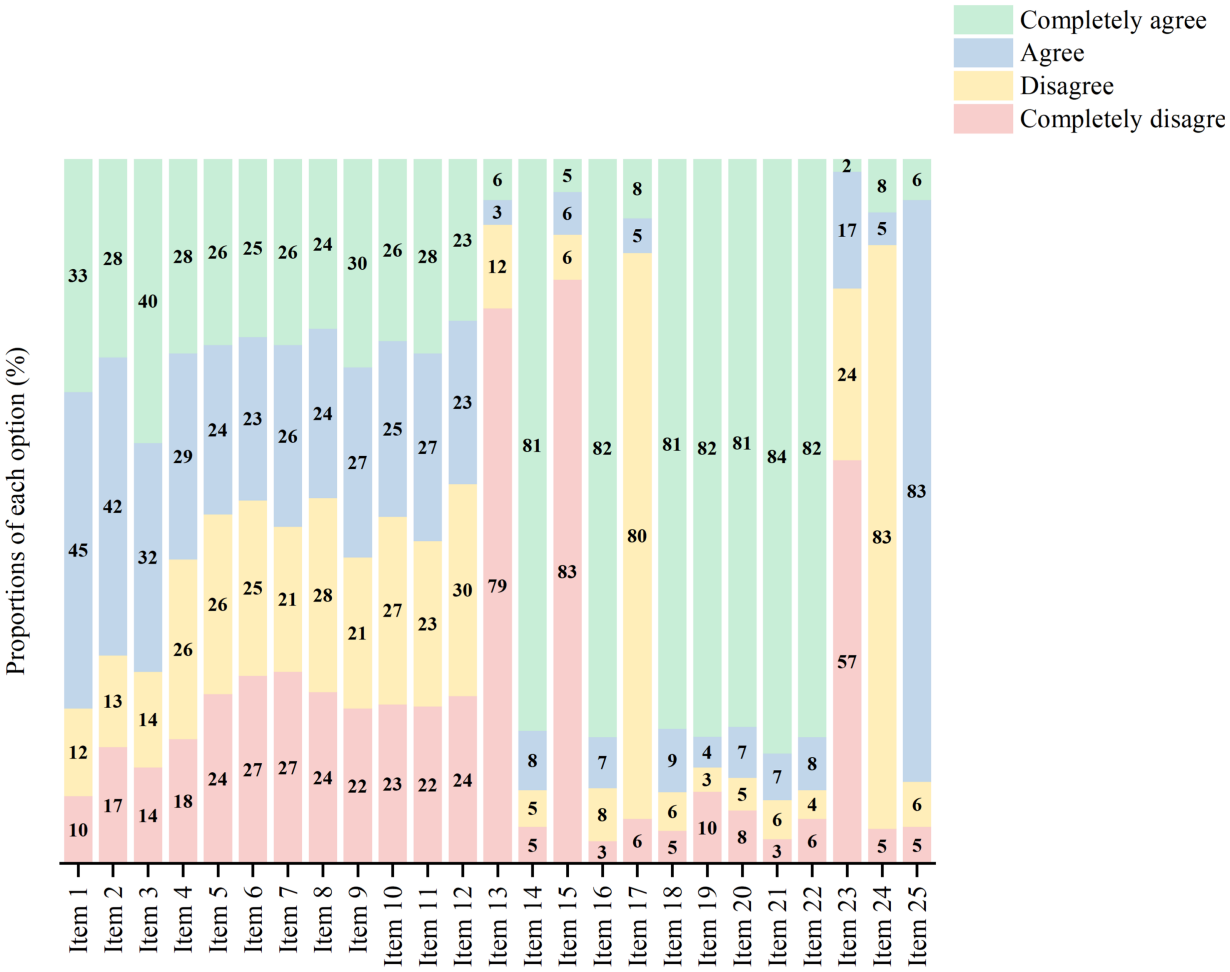
Proportions of each option on Nurses’ Attitude Survey. Detailed content of the questionnaire is provided in Supplementary file 2.

The mean attitude score of NAS was 62.86 ± 5.59. Among participants, 0.87% had low attitude scores, 86.67% were average, and 12.46% had high scores. Educational level was the only factor significantly associated with attitude scores (*p* = 0.039). Other variables, including gender, age, working unit, work experience, nursing professional level, and pain training, showed no significant associations (all *p* > 0.05). Figure 2 provides a detailed breakdown of attitude scores based on the NAS questionnaire.

### Relation between knowledge and attitude of pain management

Table 1 shows that there are significant differences in pain knowledge scores based on educational level (66.04 ± 19.86 vs. 70.49 ± 17.37 vs. 75.00 ± 9.50, *p* = 0.023) and working unit (73.65 ± 14.48 vs. 68.82 ± 18.41, *p* = 0.001), while only educational level (63.07 ± 5.61 vs. 63.07 ± 5.61 vs. 65.38 ± 3.66, *p* = 0.023) demonstrates a significant difference in attitude scores. To further analyze the relationship between knowledge and attitude of pain management, we used Spearman’s test (Table 2). The results of this test showed a weak negative correlation between knowledge and attitude scores in nurses, and the correlation was not statistically significant (*r* = −0.062, *p* = 0.248).

**Table 2:** Correlation of knowledge and attitude variables.

Variable	Mean ± SD	*r*	*p*	*n*
Knowledge score	69.94 ± 17.67	−0.062	0.248	345
Attitude score	62.86 ± 5.59			345

## Discussion

Pain is one of the most frequently reported concerns by patients, and it is essential for nurses to possess a solid understanding and a positive attitude towards its management (17). The American Pain Association has officially recognized pain as the fifth vital sign, underscoring its importance in clinical assessments (18). While pain management is a fundamental aspect of nursing care plans, it remains a complex and multifaceted issue that encompasses not only medical considerations but also legal, socioeconomic, and psychological factors. The influence of nurses’ knowledge and attitudes on effective pain management continues to be a subject of ongoing investigation.

This cross-sectional study was conducted in China, with the aim of evaluating the knowledge and attitude of emergency department nurses in the field of pain management and related factors. Our findings revealed no significant correlation between age, gender, years of experience, or professional level and pain management knowledge or attitudes, which is consistent with several previous studies (19–21). This suggests that demographic and professional characteristics may not be the primary determinants of nurses’ pain-related competencies.

Despite this, the results indicated that emergency department nurses in China have relatively insufficient knowledge and attitude toward pain management. The mean scores for the ER-SPMKQ and NAS were 69.94 ± 17.67 and 62.86 ± 5.59, respectively. Further analysis revealed that only 42.90% of the nurses demonstrated good knowledge of pain management, which is comparable to previous studies that reported 41.0 and 48.2% of nurses had good knowledge (22, 23). However, it is relatively lower than previous findings which reported 60.0, 61.1, and 66.8% of nurses had good knowledge (19, 24). Recent studies in China have also highlighted that nurses’ competencies in pain management remain suboptimal and are influenced by educational level and institutional factors (25–27). In addition, there is an increasing emphasis on competency-based frameworks and standardized assessment tools to improve clinical performance. Despite these advances, gaps between knowledge, attitudes, and actual practice persist. Several potential reasons may explain this knowledge gap. For example, several items in the ER-SPMKQ demonstrated particularly low correct response rates (Figure 1), indicating specific knowledge deficits. Notably, Item 25 assessed nurses’ perceptions regarding the proportion of patients who may over-report their pain. The low accuracy rate suggests that some nurses may overestimate the likelihood of pain exaggeration, reflecting a potential bias in pain assessment. Such misconceptions may lead to underestimation of patients’ pain and contribute to inadequate analgesic management, particularly given that pain assessment is inherently subjective and should primarily rely on patient self-report. In addition, other low-scoring items were related to opioid use and pain evaluation, further highlighting gaps in evidence-based pain management practices. Notably, a lack of specialized training in pain management, limited continuing education opportunities, and variability in pain-related content within nursing curricula may all contribute to inadequate knowledge. In our study, a significant association was found between education level and knowledge scores (Table 1), consistent with prior findings (11, 28). Higher educational attainment is often linked to more comprehensive instruction in pain management, especially in Bachelor’s or Master’s programs, compared to diploma-level training. In addition, the classification of knowledge levels based on the sample mean is a relative rather than criterion-referenced approach, which may limit comparability across studies. Future research should consider applying standardized or externally validated thresholds to improve interpretability. Furthermore, the Cronbach’s alpha values (0.716), although slightly lower than those reported in some recent studies (>0.80), remained within the acceptable range for internal consistency, which may reflect the context-specific and multidimensional nature of pain management assessment in emergency settings. Although the instruments used in this study have been previously validated, further validation in the emergency nursing context would strengthen the robustness of the findings.

Additionally, we observed that 43 nurses (12.79%) exhibited a highly positive attitude towards pain management, while 299 nurses (86.67%) demonstrated a moderate attitude. This finding contrasts sharply with previous studies, where more than half of the nurses were found to have a negative attitude towards pain management (29, 30). The observed difference may be attributed to the high proportion of nurses with a bachelor’s degree or higher in our study (85.22%). Previous studies included nurses from non-specific emergency departments and utilized different assessment tools, which could also account for these discrepancies.

The lack of specialized training in pain management may contribute to the knowledge gap observed among nurses. Further analysis indicated a significant association between education level and both knowledge and attitude scores, with higher scores correlating with increased education levels (all *p* < 0.05). This finding aligns with previous research, which has demonstrated that higher educational attainment is linked to improved knowledge and more positive attitudes toward pain management (17, 20, 23, 31). This could be attributed to the more comprehensive pain management content included in higher degree programs, such as Bachelor’s and Master’s degrees, compared to Diploma courses. Future interventions should also consider system-level factors such as workload and institutional support in addition to educational strategies.

Notably, in our study, no significant correlation was found between knowledge and attitude towards pain management (*r* = −0.062, *p* = 0.248), which contrasts with findings from previous studies (32), where higher knowledge levels were generally associated with a more positive attitude. However, some studies have suggested that knowledge and attitude may function independently (1, 21), with recent evidence reporting no significant association between these variables. One possible explanation is the presence of “moral distress,” a phenomenon in which nurses possess adequate knowledge of optimal pain management but are unable to implement it due to systemic constraints such as heavy workload, time pressure, or limited institutional support. This discrepancy between ideal practice and real-world conditions may weaken or even reverse the relationship between knowledge and attitudes. Therefore, simply increasing emergency nurses’ knowledge may not necessarily result in improved attitudes unless these broader contextual issues are also addressed.

These findings underscore the need for a multifaceted approach in improving pain management among emergency nurses. Since knowledge alone does not necessarily lead to positive attitudes, interventions in clinical practice should focus not only on education but also on addressing the unique contextual barriers faced in emergency settings—such as high workload, time constraints, and limited institutional support. Hospitals and administrators should consider implementing strategies that promote supportive environments, streamline pain management protocols, and reduce operational pressures to foster more positive attitudes. In addition, while ongoing professional training and advanced academic education are essential to enhance nurses’ knowledge base, it is equally important to monitor and mitigate any unintended negative impact on attitudes. Future programs should aim to integrate cognitive and emotional components, ensuring that knowledge acquisition is accompanied by organizational support, reflective practices, and strategies that build empathy and resilience.

### Limitation

This study has several limitations. First, the data were collected within a limited scope, and the relatively small and regionally restricted sample may limit generalizability. Although all participating hospitals were tertiary institutions in China, the findings may not be representative of nurses in other healthcare settings. In addition, convenience sampling and web-based data collection may introduce selection bias, and the lack of response rate and nonresponse analysis further limits external validity. Second, this was a cross-sectional study, which precludes causal inference. Longitudinal studies are needed to better explore the temporal relationships between knowledge and attitudes. Third, self-reported data may be subject to response bias, although confidentiality was emphasized to minimize this issue. Fourth, although the instruments used in this study have been previously validated, no additional psychometric analyses (e.g., factor analysis, test–retest reliability, or measurement invariance) were conducted, which may limit the strength of construct validity in this population. In addition, the classification of knowledge levels based on the sample mean represents a relative approach, which may limit comparability across studies. Finally, the use of unadjusted analyses without controlling for potential confounders may limit the robustness of the findings. Therefore, the results should be interpreted as exploratory, and future studies should apply multivariable and multilevel analytical approaches with larger and more diverse samples.

## Conclusion

The results of this study highlight that while emergency department nurses demonstrate a positive attitude towards pain management, their knowledge remains insufficient, indicating the need for continuous training and organized workshops to enhance pain management practices. Higher education levels significantly impact both pain management knowledge and attitudes among emergency department nurses. Additionally, nurses working in the EICU have a higher level of knowledge compared to those working in the emergency room.

## Data Availability

The original contributions presented in the study are included in the article/Supplementary material, further inquiries can be directed to the corresponding authors.
